# ALDH2 Enzyme Deficiency in Diabetic Cardiomyopathy

**DOI:** 10.3390/ijms26125516

**Published:** 2025-06-09

**Authors:** Yang-Wen Hsieh, An-Sheng Lee, Kuo-Tzu Sung, Xuan-Ren Chen, Hsin-Hung Lai, Yun-Fang Chen, Chen-Yen Chien, Hung-I Yeh, Che-Hong Chen, Chung-Lieh Hung

**Affiliations:** 1 Department of Medical Research, Mackay Memorial Hospital, Taipei City 104, Taiwan; 2 Department of Medicine, MacKay Medical College, New Taipei City 252, Taiwan; 3 Division of Cardiology, Department of Internal Medicine, MacKay Memorial Hospital, Taipei City 104, Taiwan; 4 Graduate Institute of Medical Genomics and Proteomics, College of Medicine, National Taiwan University, Taipei City 100, Taiwan; 5 Institute of Biomedical Sciences, MacKay Medical College, New Taipei City 252, Taiwan; 6 Institute of Biopharmaceutical Sciences, National Yang Ming Chiao Tung University, Taipei City 112, Taiwan; 7 Division of Cardiovascular Surgery, Department of Surgery, MacKay Memorial Hospital, Taipei City 104, Taiwan; 8 Department of Chemical and Systems Biology, Stanford University School of Medicine, Stanford, CA 94305, USA

**Keywords:** diabetic cardiomyopathy, ALDH2*2 carriers, ALDH2 enzyme deficiency, East Asian populations, ALDH2 activators, SGLT2 inhibitors

## Abstract

Diabetic cardiomyopathy (DCM) is a significant complication of diabetes, particularly affecting East Asian populations with a high prevalence of the ALDH2*2 (*Glu504Lys*) genetic variant. This variant impairs aldehyde detoxification, leading to increased oxidative stress, mitochondrial dysfunction, and chronic inflammation, exacerbating cardiac damage and fibrosis. This review aimed to systematically delineate the pathological role of ALDH2 enzyme deficiency in DCM by integrating clinical observations with mechanistic insights from experimental models and evaluating emerging therapies for genetically susceptible populations. In vitro and in vivo studies demonstrate that ALDH2*2 amplifies oxidative stress and disrupts mitochondrial homeostasis under hyperglycemic conditions, leading to enhanced cardiac fibrosis and functional decline. Additionally, ALDH2*2 carriers show heightened susceptibility to metabolic stress, further aggravating DCM. Given the high prevalence of ALDH2*2 in East Asian populations, targeted therapeutic strategies are urgently needed. Promising approaches include ALDH2 activators (e.g., Alda-1) that enhance detoxification of reactive aldehydes, and SGLT2 inhibitors (e.g., empagliflozin) that improve mitochondrial function and reduce oxidative damage. These therapies can mitigate oxidative stress and preserve cardiac function in ALDH2*2 carriers, thereby potentially reducing DCM burden, especially in high-risk East Asian populations. Further clinical investigations are warranted to validate these therapeutic approaches and optimize management for ALDH2-deficient individuals.

## 1. Introduction

Diabetic cardiomyopathy (DCM) is a specific form of heart disease seen in patients with diabetes. It is characterized by structural and functional abnormalities in the myocardium, independent of hypertension or coronary artery disease (CAD) [1,2,3]. DCM progression involves myocardial fibrosis, inflammation, oxidative stress, and mitochondrial dysfunction [4,5]. These pathological processes lead to a stiffening of the heart muscle, impaired contractility, and eventual heart failure [6]. A significant factor in DCM is the heightened oxidative stress caused by hyperglycemia, which overwhelms the heart’s antioxidant defenses [7,8].

Aldehyde dehydrogenase 2 (ALDH2) is a key mitochondrial enzyme responsible for detoxifying reactive aldehydes, including acetaldehyde, a toxic byproduct of alcohol metabolism, and 4-hydroxy-2-nonenal (4-HNE), a major lipid peroxidation product that increases under oxidative stress and diabetic conditions [9,10,11]. Through the oxidation of aldehydes to their corresponding carboxylic acids, ALDH2 plays a crucial protective role in maintaining mitochondrial function, preventing oxidative injury, and limiting cell death and fibrosis in cardiac tissues [9,10,12]. Among the ALDH2 polymorphisms, the most prevalent and clinically relevant variant is ALDH2*2 (*rs671*, *Glu504Lys*), which significantly reduces enzymatic activity. In individuals carrying this variant, aldehyde detoxification efficiency is drastically impaired, particularly under metabolic stress conditions such as hyperglycemia, thereby increasing susceptibility to cardiac injury [11,13,14].

Globally, this variant is rare in non-East Asian ancestry groups but is prevalent in East Asia, where it affects approximately 8% of the world population. In China, approximately 35–45% of the population carries this variant, while in Japan and Korea, the prevalence ranges from 30 to 40%. In Taiwan, it is even higher, reaching about 47% [15,16,17,18,19].

Many clinical studies have shown that individuals with ALDH2 deficiency are more prone to cardiovascular complications, including CAD, myocardial infarction, and heart failure [18,19,20,21,22]. In the context of diabetes, the impaired detoxification function exacerbates oxidative stress and inflammation, accelerating DCM progression [18,23].

This review aimed to systematically delineate the pathological role of ALDH2 enzyme deficiency in DCM by integrating clinical observations with mechanistic insights from experimental models and evaluating emerging therapies for genetically susceptible populations. To this end, relevant literature was retrieved from PubMed and Web of Science using the keywords “ALDH2”, “diabetic cardiomyopathy”, and “ALDH2 enzyme deficiency”, with a focus on studies published between 2000 and 2025. The majority of the studies reviewed involved human and rodent models (mice and rats), which are widely used in cardiovascular research.

## 2. Pathophysiology and Progression of DCM

DCM is often asymptomatic in its early stages, making diagnosis challenging until significant cardiac damage occurs [24,25]. As the disease progresses, patients may develop symptoms such as dyspnea, fatigue, and exercise intolerance, primarily due to impaired diastolic function that hinders the heart’s ability to relax and fill adequately [25,26]. In more advanced stages, congestive heart failure becomes evident, presenting with fluid retention (edema), orthopnea, palpitations, and chest pain, all resulting from left ventricular dysfunction. DCM is a progressive condition that begins with subtle diastolic dysfunction and eventually leads to systolic dysfunction as the heart’s contractile ability weakens. Many patients initially develop heart failure with a preserved ejection fraction; this can then progress to heart failure with a reduced ejection fraction [6,25,27].

The disease’s advancement is characterized by ventricular hypertrophy, fibrosis, and chronic inflammation, all contributing to left ventricular remodeling and increased stiffness [28]. Histologically, DCM is marked by myocardial fibrosis, with excessive collagen deposition in the interstitial and perivascular spaces, contributing to both diastolic and systolic dysfunction. Other histopathological features include cardiomyocyte hypertrophy, apoptosis, mitochondrial abnormalities, and thickened capillary basal laminas. In advanced stages, myocardial tissue may exhibit cellular disarray and necrosis, further impairing heart function. These combined pathological changes, exacerbated by oxidative stress and inflammation, underline the progressive nature of DCM, which ultimately leads to heart failure [6,25,26,29].

These pathological changes are driven by key signaling pathways, including the TGF-β1/Smad axis and the PI3K/Akt/mTOR pathway, which mediate fibrosis and mitochondrial remodeling, respectively [5,14,23].

## 3. ALDH2*2 Variant: Impaired Aldehyde Detoxification and Its Role in DCM

The ALDH2 gene encodes an essential mitochondrial enzyme that detoxifies reactive aldehydes, such as acetaldehyde—a toxic byproduct of alcohol metabolism—and 4-HNE, both of which increase under diabetic and oxidative stress conditions [9,10,11]. A common genetic variant in ALDH2, known as ALDH2*2 (*rs671*), involves a single nucleotide polymorphism on chromosome 12, where a G > A mutation leads to an amino acid substitution, Glu504Lys (E504K). This mutation drastically reduces or completely abolishes the enzyme’s activity [17,30,31]. Based on genotype, individuals can be classified as ALDH2*1/*1 (wild-type), ALDH2*1/*2 (heterozygous), or ALDH2*2/*2 (homozygous). Heterozygous individuals (ALDH2*1/*2) retain only approximately 10–20% of normal ALDH2 enzymatic activity, reflecting a substantial reduction compared to wild-type carriers. In contrast, homozygous individuals (ALDH2*2/*2) exhibit a near-complete loss of enzymatic function, with residual activity typically below 2% (Table 1). Genetically, ALDH2 deficiency encompasses both heterozygous (ALDH2*1/*2) and homozygous (ALDH2*2/*2) individuals, who exhibit partial or nearly complete loss of enzymatic activity. This substantial reduction in ALDH2 activity in ALDH2*2 carriers compromises aldehyde detoxification, increasing susceptibility to aldehyde-related health risks such as upper aerodigestive tract (UADT) cancer and cardiovascular disease [30,32,33]. The dysfunctional ALDH2 variant impacts approximately 8% of the global population. It is particularly prevalent in East Asia, affecting approximately 35–45% of the population in China, 30–40% in Japan and Korea, and 47% in Taiwan [17,18,19].

Individuals carrying the ALDH2*2 variant often experience alcohol intolerance due to impaired acetaldehyde detoxification, resulting in symptoms such as facial flushing, nausea, and palpitations. However, beyond alcohol sensitivity, the ALDH2*2 variant is associated with an increased risk of cardiovascular disease, including CAD, myocardial infarction, and heart failure [18,19,21,22]. In diabetic contexts, the reduced detoxification efficiency exacerbates oxidative stress and inflammation, accelerating DCM progression [18,23,34,35].

The cardiovascular implications of ALDH2 deficiency are profound, particularly in individuals with diabetes, as the impaired detoxification of toxic aldehydes, such as acetaldehyde and 4-HNE, exacerbates oxidative stress and inflammation [33,36,37]. In diabetic patients, this accumulation of aldehydes accelerates DCM progression. ALDH2 deficiency contributes to endothelial cell damage, inflammatory responses, and mitochondrial dysfunction, making cardiac tissue more vulnerable to ischemic injury and increasing susceptibility to heart failure [37,38,39,40]. Studies have shown that individuals with the ALDH2*2 variant not only have a higher risk for alcohol-related cancer and complications but are also more prone to diabetes-related cardiovascular diseases, including CAD and myocardial infarction, due to the cumulative effects of aldehyde toxicity and metabolic stress. Understanding the role of ALDH2*2 variants in these processes reveals therapeutic potential for targeting ALDH2 pathways, which could reduce cardiovascular risk and improve outcomes, especially in East Asian populations where ALDH2*2 prevalence is high.

ALDH2 deficiency contributes to DCM progression through multiple pathological mechanisms, including oxidative stress, mitochondrial dysfunction, inflammation, and nitric oxide (NO) dysregulation. These mechanisms collectively exacerbate cardiac fibrosis, impair myocardial function, and increase susceptibility to cardiovascular complications. Table 2 systematically illustrates how ALDH2 deficiency affects each mechanism and the corresponding pathological consequences in DCM.

## 4. Clinical Implications of ALDH2*2 Deficiency on Cardiovascular and Metabolic Health

The molecular mechanisms described above—oxidative stress, mitochondrial dysfunction, inflammation, and NO dysregulation—not only contribute to the progression of DCM in experimental models but are also reflected in clinical studies of ALDH2*2 carriers. This section reviews population-based and clinical evidence demonstrating the impact of this genetic variant on cardiovascular and metabolic health.

Studies have shown that patients with the ALDH2*2 mutation are at increased risk of cardiovascular and metabolic disorders, including CAD, heart failure, and type 2 diabetes. Individuals with AA/AG genotypes, specifically, have a higher prevalence of diabetes mellitus, indicating broader health implications than heart attacks [42,46]. The ALDH2*2 variant is linked to multiple mechanisms—such as altered alcohol metabolism, oxidative stress, and inflammation—that exacerbate traditional cardiovascular risk factors, such as high blood pressure and diabetes [42].

Additional research has found that carriers of the ALDH2*2 variant have a heightened likelihood of heart failure with preserved ejection fraction due to increased inflammation, and those with moderate alcohol consumption have poorer left atrial function, increasing the risk of atrial fibrillation [34,47]. Men with the ALDH2*2 variant who consume alcohol show higher fasting blood glucose levels, an effect not observed in those with the Glu/Glu genotype, suggesting genotype-specific glucose metabolism responses to alcohol [48].

Recent research highlights that men with the ALDH2*2 variant are at greater risk of severe heart damage during heart attacks, underscoring the need for personalized medical interventions in East Asian populations with this variant [49].

Beyond its direct role in DCM, the ALDH2*2 variant has also been extensively studied in relation to broader cardiovascular and systemic conditions. Although not specific to DCM, these associations offer valuable insights into the broader systemic impact of ALDH2 deficiency.

Research reveals that individuals carrying the ALDH2*2 variant have a higher risk of severe cardiovascular events, including myocardial infarction and macrovascular complications, regardless of alcohol intake. This variant’s effect is independent of other health factors, underscoring its role as a genetic risk factor for heart problems [50,51,52].

In a study of 202 Japanese patients with ST-segment elevation myocardial infarction, those with the ALDH2*2 variant had more frequent coronary spasms and more severe heart damage compared to non-carriers [53]. In East Asian populations, ALDH2*2 is associated with coronary spastic angina and an elevated risk of adverse cardiovascular outcomes in acute coronary syndrome [1,54].

Regarding vascular complications, individuals with the ALDH2*2 mutation are prone to higher levels of oxidative stress, reduced NO production, and impaired endothelial function, which collectively contribute to CAD [9,44].

The ALDH2*2 mutation increases acetaldehyde accumulation, which has been associated with elevated HbA1c levels, insulin resistance, and obesity in patients with type 2 diabetes, thereby implicating the variant in broader metabolic abnormalities [55,56].

The ALDH2*2 mutation has further been linked to increased risk of long-term systemic diseases, such as cancer and Alzheimer’s disease, with the influence of these risks modulated by alcohol intake [57].

Collectively, clinical studies have illustrated that individuals with the ALDH2*2 variant have a heightened risk of cardiovascular and metabolic complications—especially in East Asian populations with high variant prevalence—and this risk is exacerbated by alcohol consumption and diabetes. These findings underscore ALDH2*2’s role as a genetic risk factor for DCM and broader cardiovascular disease, warranting further investigation in experimental models.

## 5. ALDH2 Deficiency and Diabetic Cardiomyopathy: Insights from Animal and Cellular Models

While clinical evidence strongly supports ALDH2*2 as a cardiovascular risk factor, controlled animal studies help clarify the underlying mechanisms and offer insights into the impact of ALDH2 deficiency on cardiac health under diabetic conditions.

In diabetic rats, studies showed reduced heart function linked to decreased ALDH2 activity and expression. Oxidative stress-induced ALDH2 inhibition contributed significantly to heart dysfunction in these models, suggesting that ALDH2 protection could be therapeutic [23].

Studies on ALDH2-deficient mice have shown poorer heart function and increased ER stress, highlighting how ALDH2 deficiency makes the heart more vulnerable under stress [58]. This notion is further supported by studies in diabetes-related heart models, where ALDH2 activation has been shown to improve cardiac function and attenuate cardiomyocyte death, both in vivo and under high glucose conditions in vitro, without affecting systemic glucose levels [41,59].

Additionally, diabetic mice lacking ALDH2 exhibit impaired heart relaxation and reduced energy reserves, underscoring the enzyme’s role in cardiac energy regulation [60]. Another study found that low-dose ethanol treatment, which activates ALDH2, can protect diabetic rats’ hearts from ischemia/reperfusion injury by activating beneficial pathways and preventing mitochondrial damage [61].

In hyperglycemic conditions, heart damage following ischemia/reperfusion injury worsens because of increased O-GlcNAc modification of ALDH2, leading to reduced activity and extensive tissue damage [10]. In aging studies, aged ALDH2-deficient mice had a higher heart-weight-to-body-weight ratio and worse heart function than normal aged mice, suggesting that ALDH2 deficiency accelerates heart aging through increased fibrosis and oxidative stress [62].

In wild-type mice, low-dose acetaldehyde pre-treatment increased ALDH2 activity and reduced heart injury without raising acetaldehyde levels. However, in ALDH2*2 mice, the same treatment increased acetaldehyde levels and worsened heart damage [63]. Diabetic ALDH2*2 mice showed higher aldehyde accumulation, leading to mitochondrial dysfunction, apoptosis, and myocardial fibrosis [14].

Studies in ALDH2-deficient male mice reported increased weight gain, higher blood sugar, and more liver damage compared to those of female mice, suggesting a sex hormone protective effect [64]. Recently, a novel drug, AD-9308, showed promise in diabetic mice by activating an enzyme that reduces harmful compounds, such as 4-HNE, in the heart, ultimately reducing heart tissue damage and improving heart health in DCM [65].

The 2023 study by Zhang et al. [41] highlighted human serum findings, specifically noting reduced plasma ALDH2 activity in patients with diabetes. This reduction in ALDH2 activity is associated with an increased risk of DCM, underscoring ALDH2 deficiency as a critical risk factor in human diabetic populations. In streptozotocin-induced diabetic wild-type mice, ALDH2 overexpression improved cardiac function; reduced fibrosis, oxidative stress, and apoptosis; and enhanced mitophagy through the Parkin, TOM20, and Akt-GSK3β pathways. Conversely, ALDH2 deficiency in these mice exacerbated these conditions, demonstrating its protective role against mitochondrial dysfunction in diabetes [41].

These findings in animal models underscore ALDH2’s critical role in maintaining cardiac function under diabetic conditions. Cellular models allow us to delve further into specific molecular mechanisms and assess the therapeutic impact of ALDH2 modulation in a controlled environment.

Some studies concluded that ALDH2 mutations in vascular cells increase basal NO production while reducing nitroglycerin responsiveness, possibly limiting its efficacy in heart disease treatment. This mutation diminishes ALDH2 activity, leading to oxidative stress, reactive oxygen species buildup, and elevated NOS activity, all contributing to excess NO. Elevated NO levels in both in vitro and in vivo models disrupt mitochondrial function, potentially exacerbating cardiac complications through imbalanced NO signaling [45,66].

In another study, ALDH2 overexpression in heart cells exposed to high glucose improved survival rates, decreased harmful molecule accumulation, and reduced inflammation-related proteins, suggesting a potential treatment strategy against diabetes-induced cardiac damage [43].

These findings suggest that enhancing ALDH2 activity may mitigate oxidative and inflammatory responses, underscoring its therapeutic potential in DCM.

The collective findings from clinical and experimental studies underscore the role of ALDH2 deficiency as a critical genetic risk factor for cardiovascular and metabolic disease, particularly in East Asian populations [18,19,20,21,44]. Human studies have established a strong association between the ALDH2*2 variant and an increased risk of CAD, heart failure, and diabetes, regardless of alcohol consumption. Meanwhile, animal and cellular models provided mechanistic insights, demonstrating that ALDH2 deficiency aggravates oxidative stress, mitochondrial dysfunction, and inflammatory signaling pathways, leading to poorer cardiac outcomes under diabetic and ischemic conditions [10,41]. Notably, the consistent correlation between ALDH2 activity and cardioprotective effects across both human and animal models highlights the clinical relevance and translational potential of ALDH2-targeted therapeutic strategies [23,65,67]. These findings suggest that enhancing ALDH2 function—through pharmacological activators or gene therapy—may provide a viable approach for mitigating DCM and other ALDH2-related cardiovascular complications in human populations.

The presence of the ALDH2*2 variant significantly exacerbates the progression and severity of DCM, illustrating a synergistic relationship between genetic predisposition and metabolic stress [11,14,41]. Individuals with the ALDH2*2 variant exhibit diminished enzymatic detoxification of aldehydes, such as 4-HNE, leading to heightened oxidative stress, mitochondrial dysfunction, and persistent inflammatory responses. These mechanisms collectively accelerate cardiac damage and fibrosis, contributing to the structural and functional abnormalities observed in the myocardium in DCM.

This heightened vulnerability among ALDH2*2 carriers is particularly concerning in East Asian populations, where the variant prevalence is alarmingly high [16,18,34,47]. Given the increasing prevalence of diabetes in these regions, ALDH2*2 carriers are at compounded risk, underscoring the urgent need for targeted interventions.

To synthesize the clinical evidence presented above, Table 3 summarizes key studies associating the ALDH2*2 variant with cardiovascular and metabolic complications. These studies underscore the diverse pathological consequences of ALDH2 deficiency, many of which are exacerbated by alcohol consumption and metabolic stress.

**Figure 1:**
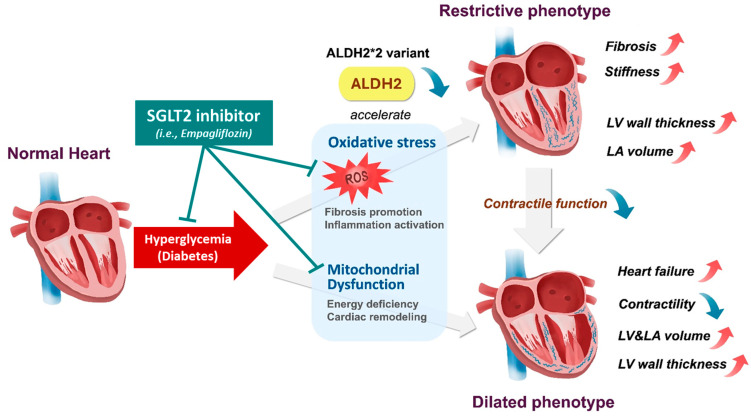
Progression of diabetic cardiomyopathy mediated by the ALDH2*2 variant and the potentiality of the SGLT2 inhibitor in alleviating relevant harmful factors in the Asian diabetic population. The diagram illustrates the progression of diabetic cardiomyopathy (DCM) mediated by the ALDH2*2 variant. Impaired detoxification due to ALDH2*2 leads to increased oxidative stress and mitochondrial dysfunction. Oxidative stress promotes fibrosis and inflammation, contributing to the restrictive phenotype with increased LV wall thickness and LA volume. Mitochondrial dysfunction causes energy deficiency and cardiac remodeling, leading to reduced contractile function and the dilated phenotype characterized by ventricular enlargement and heart failure. This pathway demonstrates how ALDH2*2 accelerates DCM progression and how implementing the SGLT2 inhibitor may be helpful, highlighting potential therapeutic targets for mitigating cardiac remodeling.

To explain this pathogenic mechanism, Figure 1 demonstrates the progression from restrictive to dilated phenotypes in DCM, emphasizing the impact of ALDH2*2-induced oxidative stress and mitochondrial dysfunction on ventricular remodeling and heart failure.

Figure 1 visually represents the transition from restrictive to dilated cardiomyopathy phenotypes, illustrating how oxidative stress and mitochondrial dysfunction, triggered by ALDH2*2, contribute to fibrosis, inflammation, and decreased contractile function. This mechanistic pathway underscores the role of ALDH2*2 in accelerating DCM progression and highlights potential therapeutic targets.

## 6. Therapeutic Directions for ALDH2*2 Carriers with DCM

Recent research has identified several promising therapeutic strategies targeting these pathological mechanisms, including ALDH2 activators, SGLT2 inhibitors, antioxidants, and anti-inflammatory agents, which collectively offer a multifaceted approach to managing DCM in ALDH2*2 carriers.

ALDH2 activators are particularly effective in addressing the root cause of oxidative stress in ALDH2*2 carriers by directly enhancing ALDH2 enzymatic activity, thereby promoting the detoxification of toxic aldehydes and reducing oxidative damage. Alda-1, a selective ALDH2 activator, decreases lipid peroxidation and myocardial apoptosis, leading to improved cardiac function post-myocardial infarction and reduced fibrosis in heart failure models [67]. Similarly, another potent ALDH2 activator, AD-9308, significantly improves diastolic and systolic myocardial function in diabetic models by enhancing mitochondrial respiration and reducing myocardial fibrosis, highlighting its potential to mitigate cardiac remodeling in DCM [65]. AD-5591 is a highly potent, selective activator of ALDH2—the key enzyme responsible for detoxifying reactive aldehydes in the body. Its prodrug, AD-9308, is water-soluble and orally bioavailable, making it ideal for in vivo application. Upon administration, AD-9308 is rapidly converted into AD-5591, which boosts ALDH2 activity, thereby reducing the accumulation of toxic aldehydes such as 4-HNE. This enhanced clearance of harmful aldehydes has been linked to improvements in both metabolic and cardiovascular conditions [70].

Complementing ALDH2 activators, SGLT2 inhibitors, such as empagliflozin and canagliflozin, exhibit cardioprotective effects beyond glucose regulation. These inhibitors improve mitochondrial function, reduce oxidative stress, and inhibit inflammatory pathways, notably the NLRP3 inflammasome, which is implicated in diabetic cardiac inflammation [11,71,72,73]. For instance, empagliflozin has been shown to enhance vascular function and mitigate cardiac remodeling in ALDH2-deficient mice by activating the AKT/eNOS pathway, thereby reducing oxidative stress [11]. In parallel, canagliflozin has demonstrated anti-ferroptotic properties, protecting cardiomyocytes against oxidative stress-induced apoptosis [72]. Intriguingly, emerging evidence suggests that SGLT2 inhibitors may also indirectly bolster cardiac resilience by upregulating ALDH2 expression [73], which could provide a dual therapeutic benefit—especially for individuals carrying the ALDH2*2 mutation associated with DCM. This dual action reduces toxic aldehyde accumulation, enhances mitochondrial function, and modulates the inflammatory response, which may lead to improved clinical outcomes in patients with DCM.

Antioxidants, including alpha-lipoic acid, N-acetylcysteine, and melatonin, provide indirect protection by scavenging reactive oxygen species and restoring ALDH2 activity. These antioxidants effectively reduce lipid peroxidation, enhance mitochondrial biogenesis, and modulate inflammatory pathways, contributing to improved cardiac function in diabetic models [9,74,75,76]. Alpha-lipoic acid has been shown to restore ALDH2 function and reduce oxidative stress in DCM [74], while N-acetylcysteine enhances glutathione levels, reducing oxidative damage, and improving cardiac function in ALDH2-deficient mice [77]. Melatonin further enhances mitochondrial function and promotes autophagy, demonstrating protective effects against diabetic cardiac apoptosis [75].

Anti-inflammatory agents are also emerging as viable therapeutic options, particularly given the central role of inflammation in DCM [70]. These agents suppress key inflammatory pathways, including the NF-κB and NLRP3 inflammasome, that contribute to myocardial fibrosis and remodeling. By inhibiting these pathways, anti-inflammatory agents reduce cardiac fibrosis and hypertrophy, thus preserving myocardial function [75,78,79]. Experimental studies have shown that NLRP3 inflammasome inhibitors effectively reduce cardiac inflammation and fibrosis, leading to improved systolic and diastolic function in diabetic models [80]. Given their potential to modulate chronic inflammation, which is a hallmark of DCM, these agents are likely to play an important role in the therapeutic landscape for ALDH2*2 carriers.

As shown in Table 4, therapeutic strategies for counteracting ALDH2 deficiency in DCM include ALDH2 activators, SGLT2 inhibitors, antioxidants, and anti-inflammatory agents. These agents target oxidative stress, mitochondrial dysfunction, and chronic inflammation, potentially improving cardiac outcomes in ALDH2*2 carriers. Table 4 summarizes their mechanisms of action, therapeutic benefits, and clinical applications, highlighting their relevance in mitigating DCM progression in high-risk populations.

Despite these promising results, challenges such as limited bioavailability, potential off-target effects, and the need for long-term safety validation remain for ALDH2-targeted therapies.

## 7. Discussion

The collective findings from clinical and experimental studies underscore the role of ALDH2 deficiency as a critical genetic risk factor for cardiovascular and metabolic disease, particularly in East Asian populations [18,19,20,21,44]. Human studies have established a strong association between the ALDH2*2 variant and increased risk of CAD, heart failure, and diabetes, regardless of alcohol consumption. These observations are mechanistically supported by experimental data demonstrating that ALDH2 deficiency aggravates oxidative stress, mitochondrial dysfunction, and inflammatory signaling pathways, leading to poorer cardiac outcomes under diabetic and ischemic conditions [10,41]. Nevertheless, many clinical studies cited in this review were conducted predominantly in East Asian populations, with factors such as alcohol consumption, sex, and age potentially introducing bias that warrants careful consideration when interpreting outcomes.

Preclinical models further highlight the cardioprotective potential of ALDH2 activity enhancement. ALDH2 activators, such as Alda-1 and AD-9308, have shown efficacy in reducing myocardial apoptosis, improving mitochondrial function, and mitigating fibrosis and oxidative damage in diabetic settings [23,65,67]. These consistent findings across animal, cellular, and human models underscore the translational relevance of ALDH2 as a therapeutic target for DCM.

Nonetheless, important gaps remain. Most clinical studies have focused on East Asian cohorts, limiting our understanding of ALDH2*2’s impact in multiethnic or global populations. Furthermore, interactions with variables such as sex, age, comorbidities, and alcohol consumption patterns require further stratified analyses. Although causal pathways are well characterized in experimental systems, human data on therapeutic modulation of ALDH2 remains sparse and warrants rigorous clinical trials.

Taken together, the ALDH2*2 variant significantly exacerbates the severity and progression of DCM, illustrating a synergistic relationship between genetic predisposition and metabolic stress [11,14,41]. Individuals with the ALDH2*2 variant exhibit diminished enzymatic detoxification of aldehydes, such as 4-HNE, leading to heightened oxidative stress, mitochondrial dysfunction, and persistent inflammatory responses. These mechanisms collectively accelerate cardiac damage and fibrosis, contributing to the structural and functional abnormalities observed in the myocardium in DCM. This heightened vulnerability among ALDH2*2 carriers is particularly concerning in East Asian populations, where the variant prevalence is alarmingly high [16,18,34,47]. Given the increasing prevalence of diabetes in these regions, ALDH2*2 carriers are at compounded risk, underscoring the urgent need for targeted interventions.

## 8. Future Directions

Future research should prioritize genotype-stratified clinical trials of ALDH2-targeted therapies to evaluate their efficacy in reducing cardiovascular risk in diabetic populations. Omics-based investigations—including transcriptomic, metabolomic, and epigenomic profiling—may further elucidate downstream regulatory networks and compensatory mechanisms in individuals with ALDH2 loss-of-function. Additionally, exploring ALDH2-related modulation in other tissues, such as liver and brain, could broaden our understanding of its systemic relevance, beyond the cardiovascular system. Further efforts are warranted to delineate tissue-specific downstream targets of ALDH2 and to clarify how sex and metabolic comorbidities may influence the therapeutic efficacy of ALDH2 modulators. As the burden of diabetes continues to rise globally, particularly in East Asian regions with high ALDH2*2 prevalence, restoring ALDH2 function represents a promising precision medicine strategy for mitigating DCM and related cardiometabolic complications.

## Figures and Tables

**Table 1:** Comparison of ALDH2 enzyme activity and related health implications by genotype.

Genotype	ALDH2 Enzyme Activity	Impact on Aldehyde Detoxification	Associated Health Risks
ALDH2*1/*1 (Wild-type)	High	Efficient detoxification of aldehydes (e.g., acetaldehyde, 4-HNE)	Lower risk of oxidative stress-related diseases
ALDH2*1/*2 (Heterozygous)	Moderate (~10–20% activity)	Reduced detoxification efficiency, leading to elevated aldehyde accumulation	Increased risk of oxidative stress, moderate vulnerability to DCM
ALDH2*2/*2 (Homozygous)	Low (<2% activity)	High aldehyde accumulation due to insufficient detoxification	High risk of DCM, coronary artery disease, and other cardiovascular diseases

**Table 2:** Mechanisms linking ALDH2 deficiency to diabetic cardiomyopathy (DCM).

Mechanism	Impact of ALDH2 Deficiency	Representative Findings	Consequence in DCM	References
Oxidative Stress	↑ 4-HNE and ROS accumulation, promoting lipid peroxidation	In diabetic ALDH2-deficient mice, increased 4-HNE levels and promoted cardiac apoptosis and fibrosis	Induces cardiomyocyte injury, enhances fibrosis, and impairs contractility	(Chen et al., 2020; Chen, et al., 2014; Guo et al., 2023; Liu et al., 2017; Wang et al., 2011) [9,10,11,18,23].
Mitochondrial Dysfunction	↓ mitochondrial membrane potential and ATP synthesis	ALDH2-deficient models showed mitochondrial swelling and reduced mitophagy under hyperglycemia	↑ myocardial energy deficiency and diastolic dysfunction	Bugger and Abel 2014; Pan et al., 2018; Tao et al., 2022; Wang et al., 2011; Zhang et al., 2023) [4,5,14,23,41]
Inflammation	Increases inflammatory cytokines (e.g., IL-6, TNF-α) and activates NLRP3 inflammasome	Cardiomyocytes under high glucose with ALDH2 inhibition displayed elevated IL-6 and NLRP3 expression	↑ chronic inflammation and myocardial remodeling	(Bugger and Abel 2014; Cao et al., 2019; Xu et al., 2014) [4,42,43]
Nitric Oxide (NO) Dysregulation	↓ NO bioavailability, ↑ NOS uncoupling, and endothelial dysfunction	ALDH2*2 variant reduced GTN efficacy and increased basal NO production via NOS uncoupling	Contributes to vascular dysfunction and left ventricular remodeling	(Chen et al., 2022; Zhu et al., 2022) [44,45]

↑, increased or upregulated; ↓, decreased or downregulated.

**Table 3:** Clinical studies linking ALDH2*2 variant to cardiovascular and metabolic diseases.

Population/ Genotype	Condition or Endpoint	Key Findings	Alcohol Interaction	References
Ethnic Asians/ALDH2*2	Atrial fibrillation (AF), left atrial strain	↑ AF risk due to alcohol-related LA dysfunction in ALDH2*2 carriers	Yes	(Hung, et al., 2021) [34]
CVD patients/ALDH2*2 carriers	Heart failure with preserved ejection fraction (HFpEF)	↑ HFpEF prevalence associated with ALDH2 rs671 polymorphism	N/A	(Tan, et al., 2020) [68]
East Asian STEMI patients	Myocardial Infarction severity, coronary Spasm	↑ ischemia/reperfusion injury and ↑ coronary spasm in ALDH2*2 carriers	N/A	(Ishida, et al., 2022) [49]
Japanese STEMI patients	STEMI, coronary spasm	↑ coronary spasms and ↑ myocardial injury in ALDH2*2 patients with STEMI	N/A	(Mizuno, et al., 2017) [69]
East Asians with ACS	GTN responsiveness, ACS prognosis	↓ nitroglycerin efficacy and ↑ adverse events in ALDH2*2 carriers	Yes	(Min and Kitakaze 2020) [54]
Japanese T2DM patients	Insulin resistance and metabolic indicators	↑ insulin resistance and obesity correlated with ALDH2*2 genotype	N/A	(Okura, et al., 2023) [56]
Japanese T2DM drinkers	Fasting glucose and HbA1c	↑ fasting glucose and HbA1c in ALDH2*2 carriers with alcohol intake	Yes	(Murata, et al., 2000) [55]
T2DM cohort/ALDH2*2 carriers	Cardio-cerebrovascular complications	↑ risk of stroke and cardiovascular events in diabetic ALDH2*2 carriers	N/A	(He, et al., 2021) [52]
East Asians/ALDH2*2 carriers	Cancer, Alzheimer’s risk	↑ long-term risk of cancer and Alzheimer’s disease; alcohol-modulated	Alcohol-modulated	(Zhao and Wang 2015) [57]
Chinese CVD patients	Myocardial infarction	↑ MI incidence in ALDH2*2 carriers in CVD cohort	N/A	(Zhu et al., 2021) [51]

↑, increased or upregulated; ↓, decreased or downregulated.

**Table 4:** Potential therapeutic strategies for ALDH2*2 carriers with diabetic cardiomyopathy.

Therapeutic Agent	Mechanism of Action	Proposed Cardioprotective Effect	Current Research Status	References
**ALDH2 Activator** Alda-1 AD-9308 AD-5591	Enhances ALDH2 activity	Reduces oxidative stress, improves cardiac function	Preclinical efficacy; early-phase trials ongoing	(Hua, et al., 2018) [67] (Lee et al., 2021) [65] (Chang et al., 2023) [70]
**SGLT2 Inhibitor** Empagliflozin Canagliflozin	Lowers glucose levels, improves endothelial function	Ameliorates hyperglycemia-induced stress, reduces CAD risk	Approved for diabetes and HF; ALDH2-specific effects under study	(Guo et al., 2023) [11] (Liu et al., 2024) [73] (Kowalska et al., 2022) [71]
**Antioxidants** Alpha-Lipoic Acid (α-LA) Dihydromyricetin (DHM) N-Acetylcysteine (NAC) Melatonin	Neutralizes ROS and reduces oxidative damage	Prevents cardiac cell apoptosis and fibrosis	Variable clinical results; more evidence needed in DCM context	(Li, et al., 2020) [74] (Chen et al., 2023) [80] (Wang et al., 2011) [23] (Rahmani et al., 2024) [76]

## Data Availability

No new data were created or analyzed in this study. Data sharing is not applicable to this article.
